# Coevolution and the Effects of Climate Change on Interacting Species

**DOI:** 10.1371/journal.pbio.1001685

**Published:** 2013-10-22

**Authors:** Tobin D. Northfield, Anthony R. Ives

**Affiliations:** Department of Zoology, University of Wisconsin, Madison, Wisconsin, United States of America; Institute of Science and Technology Austria (IST Austria), Austria

## Abstract

**Background:**

Recent studies suggest that environmental changes may tip the balance between interacting species, leading to the extinction of one or more species. While it is recognized that evolution will play a role in determining how environmental changes directly affect species, the interactions among species force us to consider the coevolutionary responses of species to environmental changes.

**Methodology/Principle Findings:**

We use simple models of competition, predation, and mutualism to organize and synthesize the ways coevolution modifies species interactions when climatic changes favor one species over another. In cases where species have conflicting interests (i.e., selection for increased interspecific interaction strength on one species is detrimental to the other), we show that coevolution reduces the effects of climate change, leading to smaller changes in abundances and reduced chances of extinction. Conversely, when species have nonconflicting interests (i.e., selection for increased interspecific interaction strength on one species benefits the other), coevolution increases the effects of climate change.

**Conclusions/Significance:**

Coevolution sets up feedback loops that either dampen or amplify the effect of environmental change on species abundances depending on whether coevolution has conflicting or nonconflicting effects on species interactions. Thus, gaining a better understanding of the coevolutionary processes between interacting species is critical for understanding how communities respond to a changing climate. We suggest experimental methods to determine which types of coevolution (conflicting or nonconflicting) drive species interactions, which should lead to better understanding of the effects of coevolution on species adaptation. Conducting these experiments across environmental gradients will test our predictions of the effects of environmental change and coevolution on ecological communities.

## Abstract 2

The effects on survival of species-species interaction in the face of a changing climate depend on whether the species' interests are conflicting or non-conflicting.

## Author Summary

Recent studies suggest that environmental changes may tip the balance between species that interact with each other, leading to the extinction of one or more species. While it is recognized that evolution will alter the way environmental changes directly affect individual species, the interactions between species force us to also consider the evolution of species interactions themselves. We use simple models of competition, predation, and mutualism to evaluate the effect of coevolution on the abundance of interacting species when climatic changes favor one species over another. In cases where the species have conflicting interests (i.e., where selection on one species for increased strength of the interaction is detrimental to the other, such as an organism becoming more aggressive towards competitors), we show that coevolution reduces the effects of climate change, leading to smaller changes in abundances and reduced chances of extinction. Conversely, when the species have nonconflicting interests (i.e., where selection for increased interaction strength on one species benefits the other, such as an organism avoiding competition with other species), coevolution increases the effects of climate change. Thus, gaining a better understanding of the nature of the coevolution between interacting species is critical for understanding how communities respond to a changing climate.

## Introduction

Climatic changes, or indeed any change in the environment, have the potential to cause the local extinction of species, and to alter community composition and ecosystem functioning [1]. Numerous models have been used to predict how the density and geographical range of species will be affected by climate change, with mixed success [2]. In part, success is limited by the need to understand how changes in the density of one species affect densities of other species through their interactions. For example, reduced pollinator densities resulting from global climate change have led to local extinction of several plant species [3]. Climate change may also have direct effects on the strength of species interactions, and these are sometimes difficult to predict [4]. For example, climatic change has been implicated in mediating extinctions of amphibian species by altering the epidemiology of their pathogens [5]. Thus, the effects of climate change on species densities and extinction risks depend both on the direct effects of climate change on focal species and on the indirect effects acting through interactions among species, making predictions of the net effect of climate change on extinction risk challenging [6]–[9].

Rapid evolution is also expected to influence species responses to climatic changes [10],[11]. In addition to evolution directly driven by the changing climate, coevolution between species may modify species interactions [12],[13]. For example, Zhang and Buckling [14] factorially manipulated the environment and a bacterium's ability to coevolve in a bacterium-phage virus system where the environmental changes reduced phage infection. The phage could persist in the presence of a changing environment or when the host was allowed to evolve, but not when both environmental change and host evolution occurred simultaneously. This phage extinction was likely caused by a combination of increased costs associated with the coevolutionary arms race and a reduced effective population size resulting from a deteriorating environment [14]. Thus, coevolution has the potential to alter species interactions to the point of reversing the fate of the interacting species and is therefore likely to be an important determinant of extinction risk and community composition [15]–[17].

The theory addressing the evolution of species in direct response to a changing climate is well known in the context of climate change (reviewed by [18]), and there is also a relevant theoretical literature addressing coevolution [19],[20]. Coevolutionary analyses of the effects of productivity on coevolving ecological communities give insights into expected community responses to climate-driven changes in densities of particular species. For example, Hochberg and van Baalen [21] used predator-prey coevolution models to show that increased prey productivity can lead to increased defense against predators and a stronger arms race. Similarly, Abrams and Vos [22] demonstrated that in some scenarios increased prey mortality can lead to increased predator density, as prey invest less in predator defense. Indeed, microcosm experiments have demonstrated that increased resource abundance for a prey species can lead to increased prey defense, resulting in lower predator-prey ratios [23],[24]. If the cost of predator defense is associated with reduced intraspecific competitive ability, selection against well-defended phenotypes is expected to be strongest when competition is strong and predation weak [25], and several empirical studies have demonstrated that this type of coevolution can drive population dynamics (e.g., [26]–[28]). Theoretical studies focused on nutrient availability and range expansion have suggested that coevolution of competitors may also alter the effects of climate change on communities. As resources decline, divergent coevolution has the potential to reduce the ratio of interspecific to intraspecific competition, leading to increased coexistence in the presence of low resource availability [29]. In cases where climate change leads to range expansion and sympatric competitor distributions, divergent coevolution can lead to increased coexistence [30].

A frequent conclusion in these and other studies is that coevolution should be stabilizing, reducing changes in population densities of interacting species (e.g., [31],[32]). Here, we examine this hypothesis in detail. Our goal is to develop a simple, general theoretical framework to organize and synthesize the ways coevolution could modify the outcome of changing environmental conditions that will likely be pervasive with climate change.

## Modeling Coevolution

To evaluate the effects of climate change, we present three coevolutionary models describing competitive, mutualistic, and predator-prey relationships between two species. Spatial structure may influence the effects of climate change on coevolving species [19],[20]; intermediate dispersal levels may slow local adaptation by diluting locally adapted genotypes, while low dispersal levels may speed local adaptation by providing advantageous genotypes [33]. In addition, when the climate itself varies across space, intermediate dispersal levels could lead to a geographic mosaic of coevolution where selection pressures and species traits vary across space [34]. Nonetheless, to focus on local adaption, we assumed that each species is represented by a single, panmictic population.

We modeled species interactions in terms of population dynamics: how the density of one species affects the population growth rate of the other. For example, for predation a high density of the predator will lead to a decrease in the population growth rate of the prey, and a high density of the prey will lead to an increase in the population growth rate of the predator; note that this general definition of predator-prey interactions encompasses host-pathogen and plant-herbivore interactions. While there is only a single interaction between species in the model, it is modeled as two parameters, one for the effect of the interaction on each species. Thus, for competitive interactions, one interaction parameter measures the negative effect of the density of the first species on the population growth rate of the second, and another parameter measures the effect of the second species on the population growth rate of the first.

We further assumed that each species has a trait that affects the strength of these interaction parameters. For example, a prey has a defensive trait that simultaneously decreases the negative effect of predation it experiences and decreases the positive effect accrued by the predator; similarly, a predator has an offensive trait that increases the predation rate on prey and increases the benefits obtained by the predator. Note that, in contrast to many models of species coevolution [35]–[37], we did not assume that there is trait matching in which the strength of interaction depends on a match between the traits values of each species; in our model both species have traits that cause monotonic benefits to the species. These benefits, however, have a cost that is exacted by decreases in the intrinsic rate of increase of the species. For example, a prey might increase its defensive trait and as a consequence suffer a reduced reproduction rate. Finally, we modeled trait evolution using a quantitative genetics approach, so the rate of evolution depends on the strength of selection and the additive genetic variance of the trait, where the additive genetic variance is constant. While this assumption about evolution is unlikely to hold in the long term (when mutations will be needed to maintain genetic variation), under very strong selection (which will cause loss of genetic variation), and for small populations (that lack large initial genetic variation and experience genetic drift), it is a reasonable starting point to investigate the short-term (hundreds of generations) response of species to climate change [38].

We used the models to pose the question: If the environment changes in such a way that the intrinsic rate of increase of one species rises, how will coevolution affect the equilibrium densities of both species? By “equilibrium density” we mean the density that would be obtained if changes in population density occurred on a more rapid time scale than evolution, although as we describe, this assumption gives insight into the case of rapid evolution on the same time scale as changes in density. We made the simplifying assumption that only one of the species experiences a direct change in its intrinsic rate of increase caused by the environmental change; this just makes it easier to separate the evolutionary changes in one species that directly experiences environmentally driven demographic changes from the other species that only responds indirectly through its interactions with the first. There is no loss of generality with this assumption, however, since the net effect of environmental changes to both species would be, to a first approximation, the simple combination of environmental changes to each species separately (Box 1).

**Box 1. Analysis of Coevolution during Climate Change**

To analyze the effects on densities and species traits that climate-driven changes in intrinsic rates of increase can have, we used an analytical approach akin to loop analysis [82]. This approach is complementary to the simulations used in the text and provides more general results that do not depend on the details of simulation models. As with loop analysis, we focus on changes in equilibrium densities with respect to changes in intrinsic rates of increase:
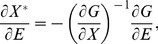
(B1)where X* = (*N*_1_*,*N*_2_*,
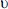
_1_*,*
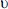
*_2_*) is the vector of the equilibrium densities and trait values for each species, *G* is a vector of functions that all equal zero when population densities and traits are at their equilibrium values (derived, for example, from Equations 1 and 2), and *∂G/∂X* is a matrix of derivatives of *G* with respect to *X*; thus, *∂G/∂X* = **A**, where **A** is a 4×4 matrix of derivatives. Assuming that environmental change *E* affects only species 1, the derivative of the equilibrium density of species 1, *N*_1_*, with respect to *E* is proportional to –cofactor(**A**,1,1)/det(**A**), where cofactor(**A**,1,1) is the determinant of matrix **A** after the first row and first column are removed.

For the cases of competition and mutualism, we can use Equation B1 to analyze the effects of conflicting versus nonconflicting evolutionary interests encapsulated in the term *d*. Formally, *d* is a partial derivative giving the change in per capita interaction strength between species with respect to the change in the other species' trait value (Text S1). Nonetheless, for simplicity we represent this partial derivative as a single term *d* that we assume is the same for both species. When *d* is small (*d* →0), the change in *N*_1_* with respect to *E* is:
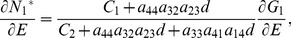
(B2)where *a_ij_* is the *ij*th element of the matrix **A**. The values *C*_1_ and *C*_2_ are positive constants such that in the absence of coevolution, 
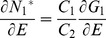
. Because for competition and mutualism (1−α^2^)*C*_1_ = *C*_2_, it follows that *C*_1_>*C*_2_. Combining this with the facts that *a*_23_ *a*_32_>0, *a*_14_ *a*_41_>0, *a*_33_<0, and *a*_44_<0, positive values of *d* increase ∂*N*_1_*/∂*E*, whereas negative values of *d* decrease ∂*N*_1_*/∂*E*. This shows that nonconflicting coevolution amplifies while conflicting coevolution dampens the effects of changing climate on population density *N*_1_* for any equation of the general form used for simulations (Equations 1 and 2).

In addition to generalizing the results from the simulations, approximation (B2) identifies the positive and negative feedback loops underlying the effects of coevolution, which are given by strings of *a_ij_*'s. The first coevolutionary loop containing *a*_32_ *a*_23_ links the evolution of the trait of species 1 with changes in the density of species 2. The second coevolutionary feedback loop containing *a*_41_ *a*_14_ links the effect of evolution of the trait of species 2 to changes in the density of species 1. Thus, the effects of evolution explicitly involve coevolution of a species trait in response to changes in the density of the interacting species. This is emphasized by the result of the approximation that if the evolution of one species has no direct effect on the density of the other (i.e., *d* = 0), evolution does not affect climate-driven changes in species densities. In other words, even if species evolve in response to climate change, and even if this evolutionary response changes the impact they experience from other species, this is not sufficient for evolution to change the response of their abundance to climate change. In addition, it is necessary for evolution of a species to affect its impact on other species it interacts with. When environmental change *E* affects both species, the derivative of the equilibrium density of species 1, *N*_1_*, with respect to *E* is equal to the sum of the derivative when only species 1 is affected and the derivative when only species 2 is affected. Thus, understanding the case where both species are affected by climate change can be easily determined by combining the cases where a single species is affected.

Finally, the same approximation approach applied to predator-prey coevolution gives the same results as for the competition and mutualism cases with *d*<0 for conflicting coevolution (Text S1).

There is a rich history of studies that show the effects of environmental change on demographic factors that affect the intrinsic rates of increase of species. For example, higher temperatures often lead to increased development rates in ectotherms, a relationship that is easily quantified [39]. Similarly, increased environmental carbon dioxide generally leads to increased plant growth, although the strength of this effect varies from species to species [40]. In addition to broad-scale climatic changes such as these, our models have implications for environmental changes on a more local scale. For example, increased nitrogen and phosphorus runoff and land management regimes can each alter growth or mortality rates, and significantly degrade the structure of ecological communities [41]–[43]. We intentionally did not specify a particular type of environmental effect in order to retain the general applicability of the models, although we recognize that there are a myriad of different effects that environmental changes can bring, and climate change will likely affect multiple environmental factors that will directly impact species' population growth rates.

A key issue in our models is how changes in the trait value of one species affects the fitness of the other species. For example, suppose that selection on the trait of a competitor to decrease the strength of competition it experiences simultaneously decreased the strength of competition experienced by the second species. This could occur if the trait reduced competition by reducing the feeding niche overlap between competitors, so the second species would benefit from selection on the first. We refer to this case as nonconflicting coevolution. Conversely, if the trait were to make the first competitor more aggressive and hence better able to defend itself against the second competitor, then the second competitor would suffer from the evolution of the first. We refer to this as conflicting coevolution. As we will show, the consequences of coevolution for the abundance of species depend on whether changes in the trait of one species is beneficial or detrimental to its interacting partner—that is, whether coevolution is nonconflicting or conflicting.

Competitors and mutualistic partners could experience either conflicting or nonconflicting coevolution, and different types of models have been used to describe each coevolutionary pathway. For example, competition models where competitors can reduce competition by shifting traits away from competitors (e.g., [35]) assume nonconflicting coevolution. In contrast, models focused on competitive arms races (e.g., [44]) assume conflicting coevolution between competitors. Although coevolution of mutualists is traditionally modeled as nonconflicting (e.g., [45]), we might expect conflicting coevolution to be common in mutualists as well. For example, yucca moths pollinate yucca plants while ovipositing in yucca flowers, and evolution of increased egg production within each flower leads to greater benefit received by the yucca moth, while negatively impacting yucca plants [46]. Thus, conflicting coevolution will occur for mutualists whenever there is the possibility of one partner cheating and reducing the benefit it provides [7],[46],[47]. For predator-prey interactions, evolution of prey to decrease the predation rate will generally be detrimental to the predator, whereas evolution of the predator to increase the predation rate will likely be detrimental to the prey. Therefore, coevolution of predator-prey interactions will generally be comparable to conflicting types of competition and mutualism, although as we discuss later, this might not strictly be the case for host-pathogen interactions.

To illustrate our theoretical results that are shared by all interactions—competition, mutualism, and predation—we used simple simulation models that share the characteristics discussed above. To aid the illustration, we selected parameter values intentionally to give coexistence of species (at least under some environmental conditions) and simple dynamics with stable equilibrium points. A theoretically more general, yet conceptually more challenging, approach to the same type of model is presented in Box 1; this general approach confirms that the qualitative patterns illustrated by our simulations are in fact found much more broadly under the general assumptions we have described.

### Competition

We modeled coevolution of two competitors using a discrete-time, modified Lotka-Volterra competition model. The density of species *i* at time *t*, *N_i,t_*, is given by
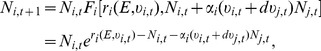
(1)in which *F_i_* gives the per capita population growth rate or, equivalently, the fitness of species *i*. The trait values that govern the strength of competition experienced by each species at time *t* are denoted 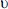 *_i_*_,*t*_ and 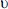 *_j_*_,*t*_. The parameter α*_i_*(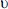 *_i_*_,*t*_+*d* 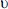 *_j_*_,*t*_) is the competition coefficient measuring the effect of species *j* on species *i*. We assumed α*_i_*(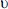 *_i_*_,*t*_+*d* 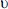 *_j_*_,*t*_) = exp(−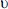 *_i_*_,*t*_−*d* 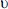 *_j_*_,*t*_). The parameter *d* determines whether coevolution is conflicting or nonconflicting, and hence is key to the model. If *d*<0, then increases in 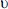 *_j_*_,*t*_ (which reduces competition experienced by species *j*) increases competition experienced by species *i*, thereby giving conflicting coevolution. Conversely, if *d*>0, then increases in 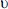 *_j_*_,*t*_ decrease competition experienced by species *i*, leading to nonconflicting coevolution. For simplicity, we assumed that the value of *d* is the same for both α_1_(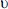
_1,*t*_+*d* 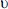
_2,*t*_) and α_2_(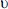
_2,*t*_+*d* 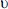
_1,*t*_), so that evolution has symmetric effects on both species.

The trait value 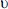 *_i_*_,*t*_ affects not only competition experienced by species *i* but also its intrinsic rate of increase *r_i_*(*E*,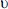 *_i_*_,*t*_). Specifically, we assumed that *r_i_*(*E*,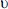 *_i_*_,*t*_) = *R_i_*+*b_i_E*−*f* 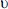 *_i_*_,*t*_, where *f* describes the cost of increasing 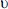 *_i_*_,*t*_; thus, there is a trade-off between reducing competition by increasing 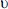 *_i_*_,*t*_ in α*_i_*(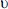 *_i_*_,*t*_+*d* 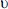 *_j_*_,*t*_) and reducing the intrinsic rate of increase, *r_i_*(*E*,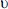 *_i_*_,*t*_). Because we assumed that α*_i_*(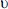 *_i_*_,*t*_+*d* 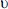 *_j_*_,*t*_) has an exponential form, there is the possibility for an optimal fitness to be achieved at intermediate values of 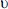 *_i_*_,*t*_. Other forms for α*_i_*(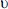 *_i_*_,*t*_+*d* 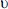 *_j_*_,*t*_) may lead to optimal fitness at either zero or infinite values of 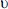 *_i_*_,*t*_; we did not consider this situation, however, because these traits experiencing disruptive selection will likely fix within a local population. Finally, we assumed that the unspecified environmental variable *E* enhances the intrinsic rate of increase of species 1 (*b*_1_>0), implying that *E* represents a more-favorable environment. For species 2, we assumed there is no effect of environmental change (*b*_2_ = 0).

In the model, 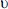 *_i_*_,*t*_ gives the mean value of a quantitative genetic trait whose distribution among individuals in the population is symmetric with additive genetic variance *V_i_*. Provided the magnitude of the variance is not too large [38],[48],[49], selection for changes in the mean value 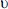 *_i_*_,*t*_ is equal to the derivative of fitness with respect to the trait divided by mean fitness [50]. For our model:
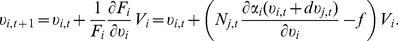
(2)


### Mutualism

The model for two mutualists has the same structure as the competition model (1). For mutualism, the coefficient α*_i_*(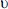 *_i_*_,*t*_, 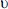 *_j_*_,*t*_) = −log(1+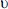 *_i_*_,*t*_+*d* 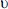 *_j_*_,*t*_) is negative, and the logarithmic form allows the optimal fitness to be achieved at intermediate values of 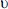 *_i_*_,*t*_. As in the competition model, *d* determines whether coevolution is conflicting or nonconflicting. The other components of the model are the same as described for the competition model, and evolutionary change is described by Equation 2.

### Predation

For predator-prey interactions we used a discrete-time version of a model in which the predator attack rate is determined by traits of both prey, 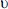
_1,*t*_, and predator, 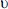
_2,*t*_ [51]. Prey trait 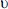
_1,*t*_ represents antipredator defense behavior, whereas predator trait 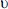
_2,*t*_ represents the ability of the predator to overcome prey defenses. Changes in the densities of prey *N_t_* and predator *P_t_* are given by:
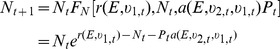
(3)

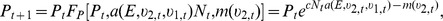
where *r* (*E*, 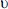
_1,*t*_) = *R*+*b_n_E*−*f* 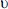
_1,*t*_ is the intrinsic rate of increase of the prey and depends on the environmental variable *E*, as in the competition and mutualism models. The predation rate *a*(*E*, 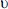
_2,*t*_, 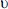
_1,*t*_) = *q*(*E*)exp(−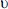
_2,*t*_ 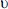
_1,*t*_) depends on the environmental variable *E* and declines with increasing prey defense, 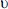
_1,*t*_, or the predator's susceptibility to the prey defense, 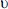
_2,*t*_. We considered two scenarios, one in which the predation rate *q*(*E*) = *Q_0_*+*b_p_E* increases linearly with *E* (*b_p_*>0) while prey growth rate is unaffected (*b_n_* = 0), and the other in which the predation rate remains constant (*b_p_* = 0) while the prey intrinsic rate of increase rises with *E* (*b_n_*>0). Although we assumed *q*(*E*) is independent of prey density for simplicity, preliminary analyses showed that incorporating a nonlinear type II functional response [52] does not qualitatively alter the results. The predator experiences a cost of trait 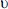
_2,*t*_ in the form of increased mortality; specifically, *m*(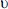
_2,*t*_) = *m*_0_+*g*/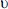
_2,*t*_, where *g* governs the cost to the predator of being able to overcome the prey defense. Finally, if *V_N_* and *V_P_* are the additive genetic variances for prey and predators, respectively, evolution is given by:
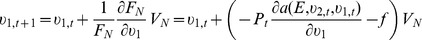
(4)

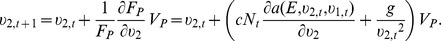



## Results

To illustrate the importance of coevolution—especially the contrast between conflicting and nonconflicting coevolution—for the response of populations to environmental changes, we conducted two types of simulations. For each type, we assumed that the populations begin at eco-evolutionary equilibrium (i.e., traits and densities are both at equilibrium), with identical genetic variances for the two species. For mutualism and competition models, the two species were initially identical in every way except in their response to environmental change. For the first type of simulation, we tracked the trajectories of population densities and traits through time as the intrinsic rate of increase of one of the species increases with the environment, *E*. We compared the trajectories for different levels of genetic variance, because the lower the genetic variance, the slower the rate of evolution. The second type of simulation involved evaluating how environmental changes alter the ecological and coevolutionary equilibriums. To find these equilibriums, after changing the environment we simulated the models for an additional 1,000 generations to allow population densities and trait values to stabilize. We did not find alternative stable states, and thus present the single equilibrium for each scenario. These two types of simulations proved to give the same conclusions, with the simulations of trajectories giving only one additional piece of information: that trait values and densities moved uniformly to the equilibriums given by the second type of simulations. The correspondence between the two types of simulations results from the fact that the level of genetic variance determines the rate of approach to equilibrium but does not alter the equilibrium itself, which is a joint optimization of fitness in each species. To avoid redundancy, we only present the trajectories for the conflicting competition case, and subsequently focus solely on the equilibrium simulations. We refer the reader to Box 1 for a full mathematical treatment that does not depend on the specific equations we used for the simulation models. Finally, although we only considered two interacting species here, we have found qualitatively similar results in simulations of larger communities (results not shown).

### Competition

**Figure 1:**
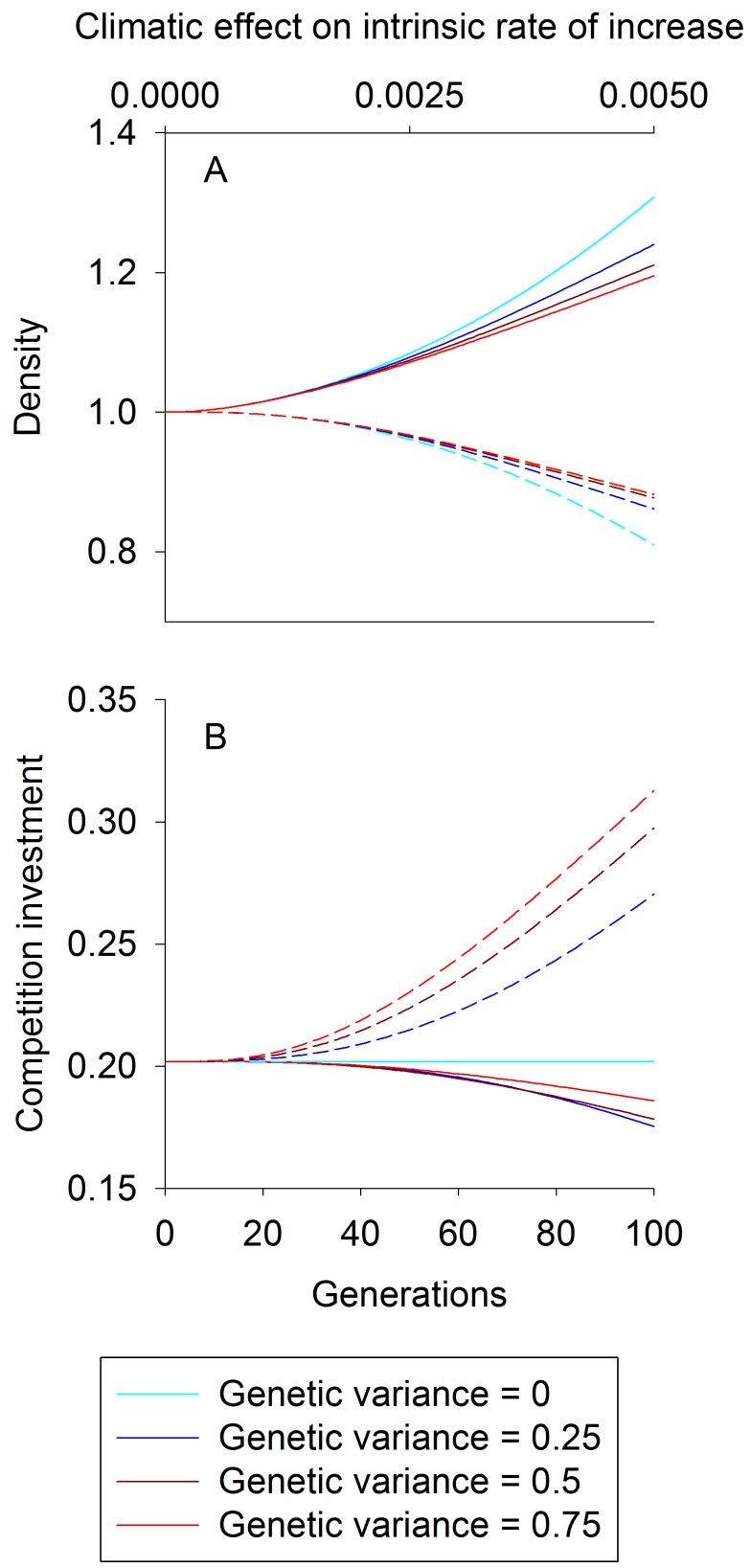
Competition density and trait trajectories. For competition, trajectories of species 1 (solid lines) and species 2 (dashed lines) densities (A) and trait values (B) as the climate variable *E* increases from 0 to 5 over the course of 100 time steps. The model describes conflicting competition (*d*<0), and additive genetic variances range from 0 (light blue) to 0.75 (red). Trajectories began at an eco-evolutionary equilibrium, and densities are scaled relative to this equilibrium. The trait value for species *i* dictates the strength of competition felt by species *i* per capita of species *j*. The top *x*-axis represents the climatic effect on the intrinsic rate of increase of species 1, *b_1_E*. Parameter values used were: *R_1_* = *R_2_* = 0.1, *d* = −0.9, *b_n1_* = 0. 001, *b_n2_* = 0, and *f* = 0.045.

To illustrate the competition model, we began by simulating the consequences of raising the environmental quality for species 1 (increasing *E*) through time while varying the rate of coevolution. When the additive genetic variances for the traits expressed by both species, *V*_1_ and *V*_2_, are zero, evolution cannot occur, whereas increasing *V*_1_ and *V*_2_ increases the rate of evolution. For this illustration we assumed competition is conflicting. Increasing *E* increases the density of species 1 and decreases the density of species 2, yet allowing evolution moderates both effects (Figure 1A). As *V*_1_ and *V*_2_ increase, the rate of change of population densities and trait values more closely track their equilibrium values N_i_* and 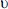 _i_*, that is, the values at which, for fixed *E*, *N_i_*_,*t*+1_ = *N_i_*_,*t*_ and 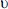 *_i_*_,*t*+1_ = 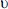 *_i_*_,*t*_ in Equations 1 and 2 (Figure 1A,B). The effects of coevolution are largely driven by changes in trait values for species 2, with less change in species 1 (Figure 1B). This occurs because species 2 evolves to invest heavily in the competitive arms race, limiting the decline in investment by species 1.

**Figure 2:**
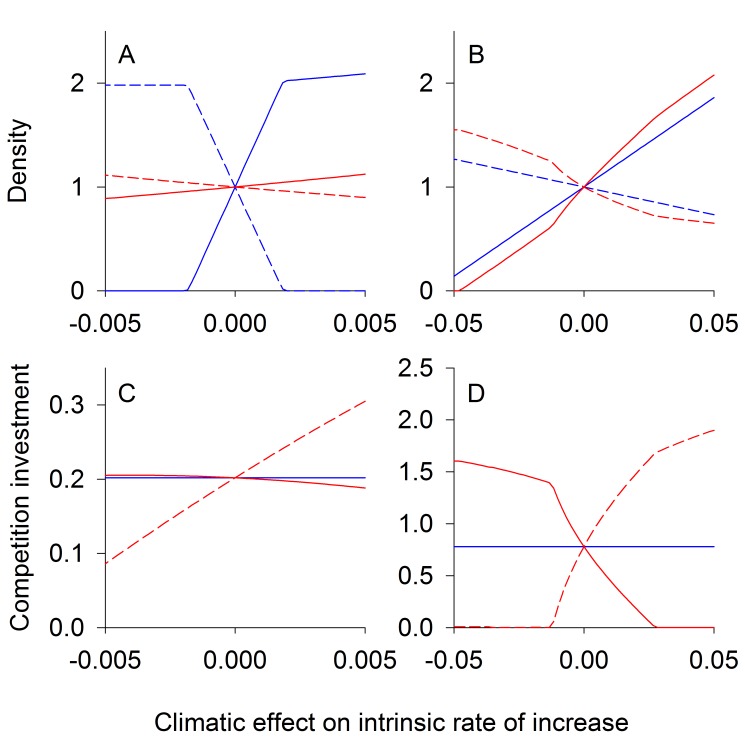
Competition equilibrium densities and traits. Equilibrium population densities (A, B) and trait values (C, D) for two competing species at different climatic conditions. The intrinsic rate of increase of species 1 (solid lines) increases linearly with climate *E*, while the intrinsic rate of increase of species 2 (dashed lines) is unaffected. Results are for conflicting competition, *d*<0 (A, C), and nonconflicting competition, *d*>0 (B, D). Red lines give eco-evolutionary equilibriums assuming high genetic variation (*V*_1_, *V*_2_>>0), whereas for blue lines there is no evolution (*V*_1_ = *V*_2_ = 0). The trait value (shown on the *y*-axis of C, D) for species *i* dictates the strength of competition felt by species *i* per capita of species *j*. The *x*-axis represents the climatic effect on the intrinsic rate of increase of species 1, *b_1_E*. For the conflicting case (A, C) parameter values used were: *R*_1_ = *R*_2_ = 0.1, *d* = −0.9, *b*_1_ = 0.001, *b*_2_ = 0, and *f* = 0.45. For the nonconflicting case (B, D) parameter values used were as follows: *R*_1_ = *R*_2_ = 0.1, *d* = 0.5, *b*_1_ = 0.01, *b*_2_ = 0, and *f* = 0.02.

For conflicting competition (*d*<0, Figure 2A,C), equilibrium species densities are less sensitive to environmental change when there is coevolution, whereas coevolution augments changes in species densities when there is nonconflicting competition (*d*>0, Figure 2B,D). This occurs because an increase in the density of species 1 with environmental change leads to a decrease in the density of species 2. Because selection pressure is positively correlated with the density of the other species, species 1 experiences relatively less selection pressure from competition with species 2 compared to the selection pressure on species 2 from species 1. When competition is conflicting (Figure 2A,C), the decreased selection on species 1 is beneficial to species 2, which acts to limit the decline of the population of species 2 and hence the decline of its effect on species 1. Also, the increased selection on species 2 increases its per capita competitive effect on species 1. These two sources of selective pressures combine to help species 2 and, in turn, are detrimental to species 1. When competition is nonconflicting (Figure 2B,D), the converse occurs; the decreased selection on species 1 caused by low densities of species 2 increases the effect of competition on species 2, and the increased selection on species 2 decreases its per capita competition effect on species 1. This selective pressure benefits species 1, further increasing its density.

In summary, conflicting competition sets up coevolution as a negative feedback, because selection on one species to reduce competition increases its competitive effect on the other species. In contrast, nonconflicting competition sets up coevolution as a positive feedback, because selection to reduce the impact of competition on one species also reduces the impact of competition on the other (Box 1).

### Mutualism

**Figure 3:**
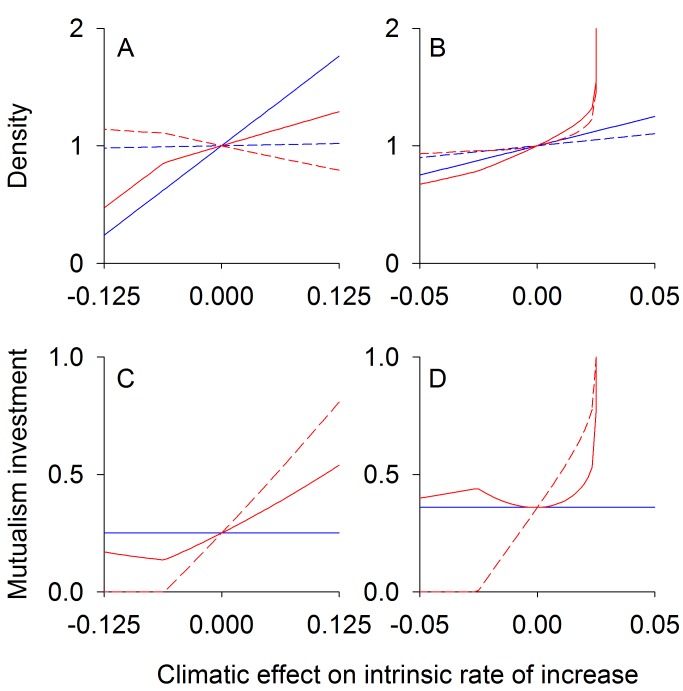
Mutualism equilibrium densities and traits. Equilibrium population densities (A, B) and trait values (C, D) under changing climatic conditions for conflicting, *d*<0 (A, C), and nonconflicting mutualists, *d*>0 (B, D). The intrinsic rate of increase of species 1 (solid lines) increases linearly with the climate variable *E*, while the intrinsic rate of increase of species 2 (dashed lines) is unaffected. The *x*-axis represents the climatic effect on the intrinsic rate of increase of species 1, *b_1_E*. Red lines give eco-evolutionary equilibriums assuming high genetic variation (*V*_1_, *V*_2_>>0), whereas for blue lines there is no evolution (*V*_1_ = *V*_2_ = 0). The trait value for species *i* (shown on the *y*-axis of C, D) dictates the benefits of mutualism accrued by species *i* per capita of species *j*. When the intrinsic rate of increase of species 1 is high enough and species are allowed to coevolve, there is no equilibrium in the nonconflicting mutualism model (B, D), as the growth of each species is unbounded. For the conflicting case (A, C) parameter values used were: *R*_1_ = *R*_2_ = 0.2, *d* = −0.9, *b*_1_ = 0.025, *b*_2_ = 0, and *f* = 0.16. For the nonconflicting case (B, D) parameter values used were as follows: *R*_1_ = *R*_2_ = 0.2, *d* = 0.4, *b*_1_ = 0.01, *b*_2_ = 0, and *f* = 0.16.

As with competition, the effects of coevolution on mutualists depended on the type of coevolution. When there is conflicting mutualism (*d*<0, Figure 3A,C), coevolution diminishes the effects of environmental change on equilibrium densities, in contrast to the case of nonconflicting mutualism (*d*>0, Figure 3B,D). This effect occurs because the increase in the density of species 1 due to the environmental change increases selection pressure on species 2 for investment in the mutualism. When the mutualism is conflicting, this change is detrimental to species 1 and limits its increase, because the benefits of mutualism decrease with the investment of species 2 in the interaction. In contrast, in the case of nonconflicting mutualism, increased investment by species 2 is beneficial to species 1, further increasing the density of species 1. In summary, conflicting mutualism sets up coevolution as a negative feedback, whereas nonconflicting mutualism sets up a positive feedback (Box 1).

### Predation

For competition and mutualism, interacting species might have either conflicting or nonconflicting coevolutionary feedbacks. In contrast, predator and prey interactions are generally expected to exhibit conflicting coevolution and hence generate negative coevolutionary feedbacks: prey coevolution of defenses that reduce predation will be detrimental to the predator, and predator coevolution to increase the predation rate will be detrimental to prey. To verify this expectation, we analyzed both the case in which climate change increases the prey intrinsic rate of increase and the case in which climate change increases the predation rate and hence the predator population growth rate.

**Figure 4:**
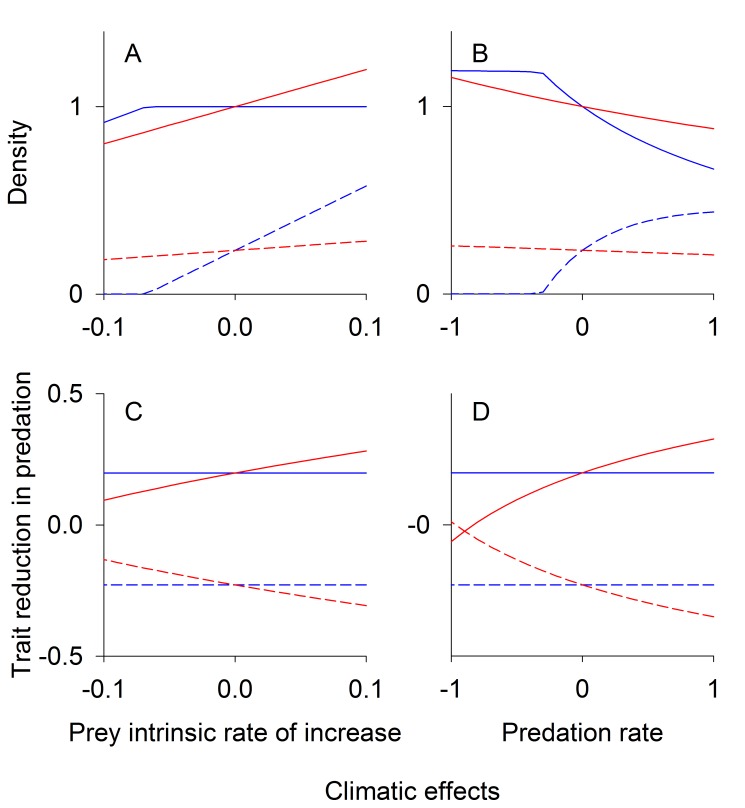
Predation equilibrium densities and traits. Equilibrium prey (solid lines) and predator (dashed lines) densities (A, B) and trait values (C, D) for different climatic conditions. Densities are scaled to equilibrium at *E* = 0. (A, C) The prey intrinsic rate of increase increases linearly with climate *E*, while the predation rate is unaffected. (B, D) The predation rate increases linearly with *E*, while the prey intrinsic rate of increase is unaffected. The *x*-axis for panels A and C represents the climatic effect on the intrinsic rate of increase of prey, *b_n_E*, and the *x*-axis for panels B and D represents the climatic effect on the predation rate, *b_p_E*. Red lines give eco-evolutionary equilibrium assuming high genetic variation (*V*_1_, *V*_2_>>0), and blue lines give the case of no coevolution (*V*_1_ = *V*_2_ = 0). Increases in either prey or predator trait values reduce per capita predation rate. Parameter values used were: *R* = 0.5, *Q*_0_ = 2, *c* = 0.25, *f* = 0.04, *g* = 0.04, and *m*_0_ = 0.005. Climate change effect parameters were either *b_p_* = 0.2 and *b_n_* = 0 (A, C) or *b_p_* = 0 and *b_n_* = 0.02 (B, D).

When climate change enhances the prey intrinsic rate of increase, the resulting increase in prey density leads to increased predator density, and in the absence of coevolution the equilibrium predator density increases dramatically (Figure 4A). In contrast, the equilibrium predator density increases more slowly when predator and prey coevolve (Figure 4A). As with conflicting competition and mutualism, higher predator density strengthens selection pressure for prey investment in the coevolutionary arms race (Figure 4C). With increased prey investment, the predator density cannot increase as much due to heightened prey defense (Figure 4A,C).

When climate change increases the predation rate but has no effect on the prey intrinsic rate of increase, the predator density increases rapidly in the absence of coevolution, but this increase is slowed by coevolution (Figure 4B,D). Because prey selection pressure is positively correlated with predator density, prey evolve higher defensive trait values in the presence of higher predation rates, which in turn lowers the predation rate, increases prey density, and decreases predator density. Thus, coevolution sets up a negative feedback loop that reduces the decline in prey density and increase in predator density (Figure 4B). While these results pertain to specialist predators that have no other prey species, we found that coevolution also reduces the ecological effects of climate change in a model for generalist predators (Figure S1).

## Discussion

We have shown, using simple models, that coevolution may increase or decrease the effect of environmental change, depending on the form that coevolution takes between species. In cases where species have conflicting interests, coevolution reduces the effects of environmental change on densities, because coevolution acts as a negative feedback to the effects of environmental change. Conversely, when species have nonconflicting interests, coevolution sets up a positive feedback that increases the effects of environmental change on densities. Given these contrasts, is coevolution in nature likely to involve conflicting or nonconflicting interests of interacting species? Competitors and mutualists, in particular, have the potential to coevolve along either conflicting or nonconflicting pathways. Thus, determining the predominant type of coevolution will be critical to identifying the long-term effects of climate change on species.

Below, we first give brief discussions of classical studies and show that cases of both conflicting and nonconflicting coevolution are common. Therefore, no *a priori* prediction can be made for their relative importance when anticipating the effects of climate change. We then turn to coevolutionary studies that directly address climate change, using these to show how evidence can be obtained to make and test predictions about the coevolutionary effects on specific systems facing climate change.

### Competitive Coevolution

It has long been recognized that coevolution can lead to increased asymmetries in competitive abilities [15], which is the hallmark of conflicting coevolution. But the idea that competition drives partitioning of food sources is even older [53]–[55], and this is the hallmark of nonconflicting coevolution. The effects of climate change for specific competitors hinge on which type of coevolution occurs. Evidence suggests that both are common.

Laboratory experiments that evaluate the effect of competitive interactions on trait evolution for each species have documented both conflicting coevolution in flies [15] and nonconflicting coevolution in *E. coli* strains [56]. Furthermore, conflicting and nonconflicting coevolution are not mutually exclusive; *Colpoda* protozoans with initially weak competitive abilities have been shown to evolve along both pathways [57]. While these types of experimental studies have the advantage of documenting coevolution as it happens, they are limited by the range of species and time scales that are amenable to experiments, and the magnitude of environmental heterogeneity that may affect coevolution [58],[59].

Alternatively, field studies can be used to infer the prevalence of conflicting versus nonconflicting coevolution. Research focusing on character displacement in natural populations attempts to identify the effects of coevolutionary processes based on species' phenotypes in solitary and sympatric populations [60]. This approach has documented both conflicting [61],[62] and nonconflicting coevolution [63]–[65].

### Mutualistic Coevolution

There is a rich theory describing the evolution of mutualisms [66],[67]. Theoretical predictions often suggest that mutualistic interactions have the potential to break down into parasitic interactions [47],[68],[69]; this is an extreme form of conflicting interests between species. If mutualism breakdown into parasitism is common, then conflicting coevolution is likely, and this will likely diminish the effects of climate change.

Nonetheless, if mutualistic partners can enforce good behavior of their partners [68], then nonconflicting coevolution is expected. For example, the plant *Medicago truncatula* discriminately rewards the most beneficial mycorrhizal partners with more carbohydrates, and mycorrhizal partners form partnerships only with the roots that provide the most carbohydrates [7]. Thus, each partner constrains the selection pressure of the other to allow only nonconflicting coevolution. If nonconflicting coevolution is frequently imposed by mutualists, our results suggest that coevolution between mutualistic species will exaggerate, rather than diminish, the effects of climate change on species densities.

### Predator-Prey Coevolution

Conflicting coevolution is expected for most types of predator-prey or consumer-resource interactions, because increases in prey defenses will decrease benefits to predators, and increases in predator effectiveness will be detrimental to prey. Nonetheless, evolution of parasite virulence could be different [70],[71]. The conventional wisdom is that parasites should evolve to be less virulent, because this will increase their transmission among hosts; parasites are not transmitted by dead hosts, at least not for long [72]. Nonetheless, this ignores, among other things, the relationship between the production of large numbers of propagules (that generally harms the host) and transmission rates, and more-detailed analyses generally predict evolution of parasite virulence to represent a balance between higher virulence caused by selection for production of propagules and lower virulence caused by selection for lengthening the transmission period [73]. Therefore, evolution of the parasite may be nonconflicting with the host, even at the same time evolution of the host to limit infection is conflicting with the parasite. In models describing this interaction (results not shown), we found that when climatic changes directly affect the parasite, coevolution in the host fuels a negative feedback loop that mitigates the effects of climate change. In contrast, in some cases when climatic changes directly affect the host, coevolution can lead to a positive feedback loop that exaggerates the effects of climate change on the host density. Thus, when there are both conflicting and nonconflicting coevolution, the ultimate outcome will be determined by whether the host or parasite experiences greater evolutionary change.

### Predicting the Effects of Climate Change

Given the widespread occurrences of both conflicting and nonconflicting coevolution in competition and mutualism, and to a lesser extent in predator-prey interactions, systems will have to be studied on a case-by-case basis to predict and test the role of coevolution in modifying the effects of climate change. This could be done either using experimental studies or taking advantage of naturally occurring environmental gradients.

An example of an experimental study is given by Lopez-Pascua and Buckling [74], who performed an environmental manipulation of bacterial productivity by altering nutrient concentrations in the growth media. They showed that increasing bacterial productivity increases the rate of coevolution between bacteria and phages. They proposed that this is due, in part, to increased selection pressure on the bacterial population in environments with high productivity (high intrinsic rates of bacterial increase). This increased selection stems from increased encounters with phages, as phages numerically respond to increased bacterial density. The phages then evolve greater infectivity in response to bacterial evolution. This explanation is consistent with our theoretical expectations for conflicting evolution of prey and predators; increasing the prey intrinsic rate of increase leads to evolution of stronger prey defenses against the predator (Figure 4C).

In addition to experimental manipulations of environmental factors, it is possible to take advantage of natural environmental gradients similar to classical studies of character displacement. For example, in a field experiment, Toju et al. [75] documented a climatic gradient in a coevolutionary arms race between the camellia beetle (*Curculio camelliae*) and its host plant, Japanese camellia (*Camellia japonica*). Female beetles use their snout to pierce the camellia fruit pericarp and oviposit eggs into seeds, with oviposition success determined by the length of the beetle's snout and ovipositor relative to the pericarp thickness. Thus, plant defense is determined by pericarp thickness, and beetle snout and ovipositor lengths determine beetle ability to overcome this defense. The authors measured beetle and plant traits along a latitudinal gradient, and previous work had showed that plants exhibit faster potential for growth at lower latitudes [76]. Our analyses suggest that, because increases in prey growth should increase predator densities and, in turn, increase selection pressure on prey, the coevolutionary arms race should be “won” by prey under environmental conditions that favor prey population growth (Figure 4A,C). Thus, in the camellia-beetle arms race we expect that coevolution will favor plants more at lower latitudes. The authors indeed found this to be the case; plants in high latitude populations that experienced endemic predation by beetles had pericarp thicknesses similar to populations that did not experience beetles. In contrast, at lower latitudes plant populations that experienced beetle predation had thicker pericarps than populations that did not. There was thus an increase in plant defense along the environmental gradient. Furthermore, this plant defense increased with decreasing latitude at a greater rate than weevil ovipositor length, suggesting that plants exhibited a larger coevolutionary advantage in environmental conditions with increased prey growth [75]. These results support our theoretical predictions that higher prey intrinsic rates of increase should lead to a coevolutionary advantage to prey, thereby buffering the changes in predator densities driven by climate change.

The majority of coevolutionary studies involving environmental manipulations or environmental gradients have been conducted on predator-prey or herbivore-plant systems where conflicting coevolution is likely. Similar experiments that document changes in traits and density might help build a better understanding of coevolution in competitive and mutualistic relationships. Laboratory studies have suggested that coevolution can lead to a reversal of competitive hierarchy in just 24 generations [15], and can occur fast enough to drive population dynamics [16]. Therefore, experimental competition studies in which environmental factors are manipulated are possible for some types of organisms. Environmental gradient, rather than experimental, studies will be more practical for larger organisms with longer lifespans that operate at larger spatial scales. Using character displacement to infer conflicting versus nonconflicting coevolution is necessarily correlative, although it opens up the study of coevolution in the context of climate change to a much wider range of species under natural spatial and temporal scales.

Studies that evaluate coevolution over environmental gradients fit within the broader conceptual paradigm of geographic mosaic theory [77] in which differences in coevolutionary selection among spatially separated populations are analyzed as genotype by genotype by environment interactions. A key feature of geographic mosaic theory is that some local populations experience environmental conditions under which coevolutionary pressures are strong. These “coevolutionary hotspots” are characterized by fitness equations ([77], p. 100)
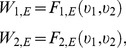
(5)where the fitnesses *W*_1,*E*_ and *W*_2,*E*_ of species 1 and 2 depend on both phenotypes 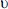
_1_ and 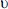
_2_, and on the environment *E*. This pair of equations has the same general structure as that we have used for Equations 1–4. Thus, our results address the possible character of evolution within coevolutionary hotspots, and how coevolutionary outcomes might differ under different environmental regimes.

We have only considered local populations, explicitly ignoring gene flow among populations. Thus, we have ignored the large body of theoretical and empirical studies evaluating gene flow among populations under different selective forces [77]–[79]. For example, Nuismer et al. [80] used spatially explicit population genetics models to show that isolated populations of mutualistic species were likely to reach equilibrium quickly, while antagonistic populations were likely to oscillate in both density and phenotype. When interaction types vary spatially, however, both dynamic and equilibrium clines occur, and the presence of each depends on the levels of selection and gene flow across the landscape [80]. In an experimental bacteria-bacteriophage community, bacteriophages became locally maladapted in the absence of gene flow, but became locally adapted when gene flow occurred between bacteriophage populations [81]. The importance of gene flow in both theoretical and empirical studies gives a caution to our recommendation that natural environmental gradients be used to assess the character of coevolution—conflicting versus nonconflicting—and whether coevolution sets up positive or negative feedback loops to environmental changes. Gene flow and a geographic mosaic of selective pressure may dampen or otherwise modify the effects of local selection on coevolutionary traits.

### Conclusions

While it is recognized that evolution will play a role in determining how climatic changes directly affect species [18], the interactions among species force us to also consider coevolution between species. Our models suggest that the effects of coevolution on population densities depend on the presence of conflicting versus nonconflicting coevolutionary interests. While we encourage future studies that experimentally manipulate both coevolution and environmental change, we acknowledge that experiments are likely to be difficult logistically for most study systems. It may be possible, however, to use character displacement across environmental gradients to distinguish whether conflicting versus nonconflicting coevolution is more likely, even when directly measuring coevolution is impossible.

Experimental [15] and environmental gradient [60] approaches to infer the nature of coevolution are both five decades old, and we hope that our theoretical results provide new impetus for these types of studies. They give needed information to anticipate whether coevolution will increase or decrease the effects of climate change on the densities of interacting species.

## Supporting Information

Figure S1 **Generalist predator equilibrium densities and traits.** Equilibrium values of prey and generalist predator population densities (A, B) and traits (C, D) for different climatic conditions. Densities are scaled to the prey equilibrium density at *E* = 0. (A, C) The prey intrinsic rate of increase rose linearly with climate *E*, while the predation rate was unaffected. (B, D) The predation rate increased linearly with climate *E*, while prey growth was unaffected. Red lines give eco-evolutionary equilibrium assuming high genetic variation (*V*_1_, *V*_2_>>0), and blue lines give the case of no coevolution (*V*_1_ = *V*_2_ = 0). Parameter values used were: *R_n_* = 0.5, *R_p_* = 0.2, *Q*_0_ = 2, *c* = 0.25, *f* = 0.04, *g* = 0.04, and *m*_0_ = *P_t_*−0.2 (to account logistic growth on alternative resources). Climate change effect parameters were either *b_p_* = 0.2 and *b_n_* = 0 (A, C), or *b_p_* = 0 and *b_n_* = 0.02 (B, D).
(TIF)

Text S1 **Analytical approximation for changes in species abundances with coevolution.**
(DOC)
