# Supplementary figures and images for: Coevolution and the Effects of Climate Change on Interacting Species
Source: PLoS Biol. 2013 Oct 22;11(10):e1001685. doi: 10.1371/journal.pbio.1001685 (PMC3805473; doi:10.1371/journal.pbio.1001685)

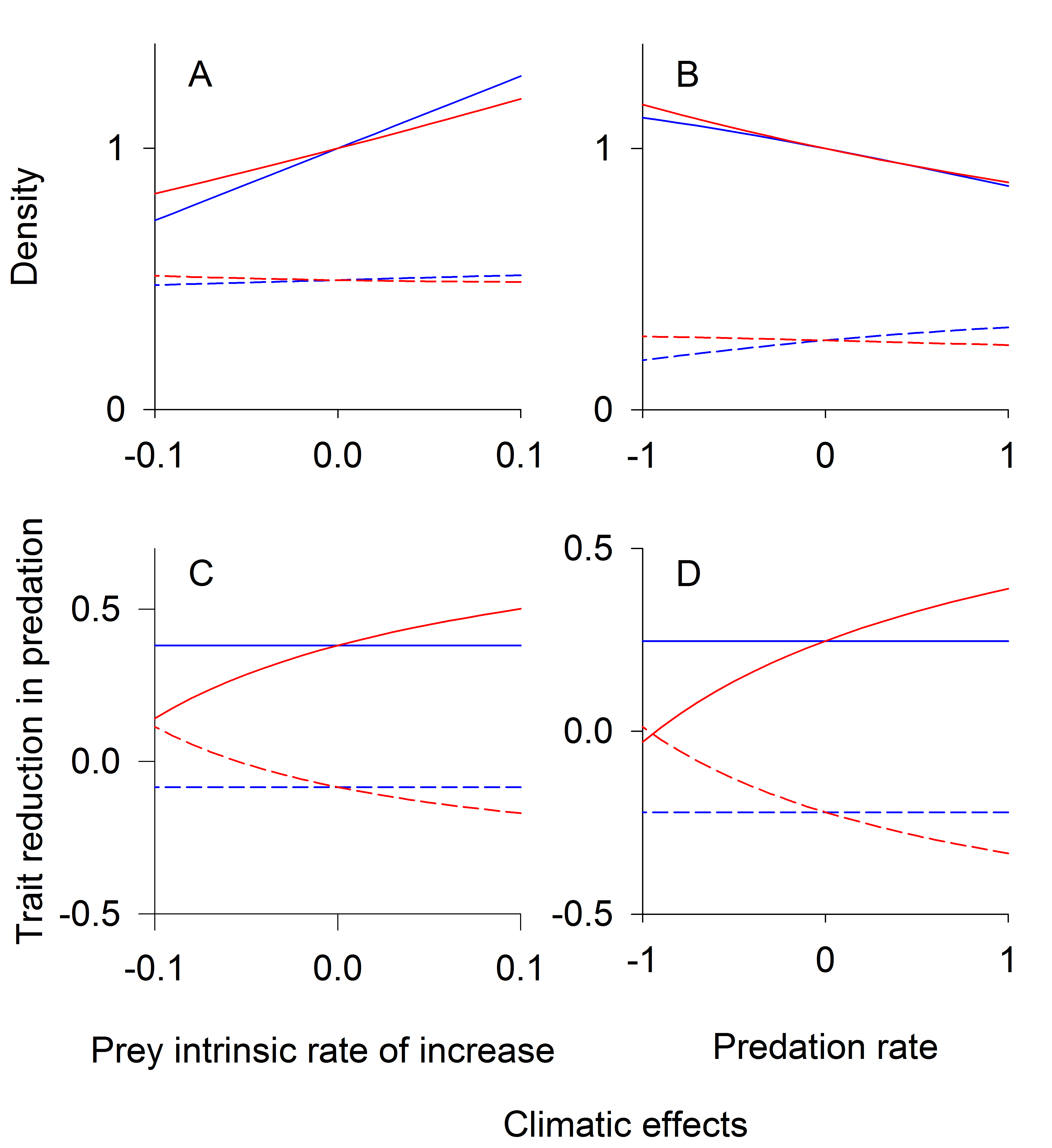

Supplement: Figure S1 — Generalist predator equilibrium densities and traits. Equilibrium values of prey and generalist predator population densities (A, B) and traits (C, D) for different climatic conditions. Densities are scaled to the prey equilibrium density at E = 0. (A, C) The prey intrinsic rate of increase rose linearly with climate E, while the predation rate was unaffected. (B, D) The predation rate increased linearly with climate E, while prey growth was unaffected. Red lines give eco-evolutionary equilibrium assuming high genetic variation (V 1, V 2>>0), and blue lines give the case of no coevolution (V 1 = V 2 = 0). Parameter values used were: Rn = 0.5, Rp = 0.2, Q 0 = 2, c = 0.25, f = 0.04, g = 0.04, and m 0 = Pt−0.2 (to account logistic growth on alternative resources). Climate change effect parameters were either bp = 0.2 and bn = 0 (A, C), or bp = 0 and bn = 0.02 (B, D). (TIF) [file pbio.1001685.s001.tif]
