# Supplementary material for: Coevolution and the Effects of Climate Change on Interacting Species
Source: PLoS Biol. 2013 Oct 22;11(10):e1001685. doi: 10.1371/journal.pbio.1001685 (PMC3805473; doi:10.1371/journal.pbio.1001685)
Supplement: Text S1 — Analytical approximation for changes in species abundances with coevolution. (DOC) [file pbio.1001685.s002.doc]

**Text S1. Analytical approximation for changes in species abundances with coevolution.** The analyses presented in the Box in the main text can be used to both generalize the results from the simulations and expose the causal pathways underlying the role of coevolution in determining the changes in species abundances when climate change alters the intrinsic rate of increase of one species. Consider the general model

*N*_1_,*_t_*_+1_ = *N*_1_,*_t_F*_1_(*N*_1_,*_t_*, α_1_(υ_1_*_,t_*, υ_2_*_,t_*)*N*_2_,*_t_*, υ_1_,*_t_*)

*N*_2_,*_t_*_+1_ = *N*_2_,*_t_F*_2_(*N*_2_,*_t_*, α_2_(υ_1_*_,t_*, υ_2_*_,t_*)*N*_1_,*_t_*, υ_2_,*_t_*) . (S1)

This structure is an obvious generalization of the competition/mutualism model. It also has the same structure as the predator-prey model; if *N*_1_ is the prey and *N*_2_ is the predator, then α_1_(υ_1_, υ_2_) = *a*(*E*, υ_1_, υ_2_), and α_2_(υ_1_, υ_2_) = –*a*(*E*, υ_1_, υ_2_)*c*, where *c* is the predator conversion rate.

From the Implicit Function Theorem,

$\frac{\partial X*}{\partial E}=-\left(\frac{\partial G}{\partial X}\right)^{-1}\frac{\partial G}{\partial E}$ (S2)

where *X**=(*N*_1_*,*N*_2_*,υ_1_*,υ_2_*) is the vector of the equilibrium densities and trait values for each species, and *G* is a vector of functions that all equal zero when population densities and traits are at their equilibrium values; specifically,

*G*_1_(*N*_1_, α_1_(υ_1_, υ_2_)*N*_2_, υ_1_) = *F*_1_ – 1

*G*_2_(*N*_2_, α_2_(υ_1_, υ_2_)*N*_1_, υ_2_) = *F*_2_ – 1

*G*_3_(*N*_1_, α_1_(υ_1_, υ_2_)*N*_2_, υ_1_,[D_1_α_1_]*N*_2_) = $\frac{dF_{1}}{d\upsilon_{1}}\frac{V_{1}}{F_{1}}$

*G*_4_(*N*_2_, α_2_(υ_1_, υ_2_)*N*_1_, υ_2_,[D_2_α_2_]*N*_1_) = $\frac{dF_{2}}{d\upsilon_{2}}\frac{V_{2}}{F_{2}}$

where the notation [D*_i_f*] denotes the partial derivative of *f* with respect to variable *i*; hence, [D_1_α_1_] = ∂α_1_/∂υ_1_. Letting *∂G/∂X* = **A**, and assuming that *E* affects only species 1, the change in the sum of species abundances with respect to *E* is

$\frac{\partial\left(X_{1}*+X_{2}*\right)}{\partial E}=-\frac{\text{cof}(\mathbf{A},1,1)+\text{cof}(\mathbf{A},2,1)}{\det(\mathbf{A})}\frac{\partial G}{\partial E}$ (S3)

where cof(**A**,*i*,*j*) = (–1)*^i^*^+^*^j^* det([**A**]*_i_*,*_j_*) and [**A**]*_i_*,*_j_* is matrix **A** with the *i*^th^ row and *j*^th^ column removed.

The elements of **A** can be derived for *G*_1_ and *G*_3_ as

*a*_11_ = [D_1_*G*_1_] = –1

*a*_12_ = [D_1_*G*_1_]α_1_

*a*_13_ = [D_2_*G*_1_][D_1_α_1_]*N*_2_ + [D_3_*G*_1_] = 0

*a*_14_*d*_1_ = [D_2_*G*_1_]*N*_2_[D_2_α_1_]

*a*_31_ = [D_1_*G*_3_]

*a*_32_ = [D_2_*G*_3_]α_1_ + [D_4_*G*_3_][D_1_α_1_]

*a*_33_ = [D_2_*G*_3_]*N*_2_[D_1_α_1_] + [D_3_*G*_3_] + [D_4_*G*_3_][D_11_α_1_]*N*_2_

*a*_34_*d*_1_ = [D_2_*G*_3_]*N*_2_[D_2_α_1_] + [D_4_*G*_4_][D_12_α_1_]*N*_2_,

with symmetric equations for *G*_2_ and *G*_4_. The term *d*_1_ represents the cross derivatives [D_2_α_1_] and [D_12_α_1_] that show when the interaction effect from species 2 on species 1, α_1_(υ_1_, υ_2_), changes with the trait value of species 2. In the specific equations we use for competition and mutualism in which α_1_(υ_1_, υ_2_) = α_1_(υ_1_ + *d*υ_2_), *d* is synonymous with *d*_1_.

Expanding equation (S3) in terms of *a_ij_* and excluding terms of order *d_i_*^2^ yields

$$\text{cof}(\mathbf{A},1,1)+\text{cof}(\mathbf{A},2,1)=\left(-1-a_{21}\right)a_{33}a_{44}-a_{23}a_{32}a_{44}d_{2}+a_{23}a_{31}a_{44}d_{1}$$

$$\det(\mathbf{A})=\left(1-a_{12}a_{21}\right)a_{33}a_{44}+d_{2}\left(a_{23}a_{32}a_{44}+a_{23}a_{31}a_{12}a_{44}\right)+d_{1}\left(a_{14}a_{41}a_{33}+a_{14}a_{42}a_{21}a_{33}\right)$$

(S4)

Note that when *d*_1_ = *d*_2_ = 0,

$\frac{\partial\left(X_{1}*+X_{2}*\right)}{\partial E}=-\frac{\left(-1-a_{21}\right)a_{33}a_{44}}{\left(1-a_{12}a_{21}\right)a_{33}a_{44}}\frac{\partial G}{\partial E}=\frac{\left(1+a_{21}\right)}{\left(1-a_{12}a_{21}\right)}\frac{\partial G}{\partial E}$ (S5)

which is identical to the result in the absence of coevolution. This shows that coevolution affects the response of equilibrium abundances to changes in *E* only when selection on the trait values of one species changes its interactive effects α*_i_*(υ_1_, υ_2_) on the other species.

Letting *C*_1_ = (1 + *a*_21_)*a*_33_*a*_44_, *C*_2_ = (1 – *a*_12_*a*_21_)*a*_33_*a*_44_,

$\frac{\partial X_{1}*+X_{2}*}{\partial E}=\frac{C_{1}+a_{32}a_{23}a_{44}d_{2}-a_{31}a_{23}a_{44}d_{1}}{C_{2}+\left(a_{23}a_{32}a_{44}+a_{23}a_{31}a_{12}a_{44}\right)d_{2}+\left(a_{14}a_{41}a_{33}+a_{14}a_{42}a_{21}a_{33}\right)d_{1}}\frac{\partial G_{1}}{\partial E}$ (S6)

This expression gives a straightforward way of generalizing the simulation results for the specific forms of competition, mutualism, and predator-prey equations we used. For example, for competition

$\frac{\partial X_{1}*+X_{2}*}{\partial E}=\frac{\left(1-\alpha_{2}\right)\alpha_{1}\alpha_{2}N_{1}N_{2}+\alpha_{1}\alpha_{2}^{2}N_{1}^{2}d}{(1-\alpha_{1}\alpha_{2})\alpha_{1}\alpha_{2}N_{1}N_{2}+\alpha_{1}\alpha_{2}(\alpha_{1}N_{2}^{2}+\alpha_{2}N_{1}^{2})d}\frac{\partial G_{1}}{\partial E}$ (S7)

Because existence of a positive stable equilibrium requires |α*_i_*| < 1, it follows that ∂(*X*_1_*+ *X*_2_*)/∂*E* increases with *d*, implying that non-conflicting coevolution will always increase the change in summed equilibrium abundances with changes in *E*.

Broader generalities can be obtained directly from equation (S6) by noting constraints on the signs of the terms *a_ij_*. The cases of competition and mutualism are similar, as follows. For traits to have optimal values, *a*_33_ < 0 and *a*_44_ < 0. If higher trait values *v_i_* reduce the interaction impact on species *i* from the other species (as in our models), then *a*_23_*a*_32_ > 0 and *a*_14_*a*_41_ > 0. For the exponential forms of equations that we used for simulations, i.e., *F*_1_(*N*_1_, α_1_*N*_2_, υ_1_) = exp(*f*_1_(*N*_1_, α_1_*N*_2_, υ_1_)), the terms *a*_13_ = *a*_24_ = 0. Assuming that these values are small, equation S6 simplifies to

$\frac{\partial X_{1}*+X_{2}*}{\partial E}=\frac{C_{1}+a_{32}a_{23}a_{44}d_{2}}{C_{2}+a_{23}a_{32}a_{44}d_{2}+a_{14}a_{41}a_{33}d_{1}}\frac{\partial G_{1}}{\partial E}$ (S8)

For competition and mutualism, *C*_1_ > *C*_2_, which leads immediately to the conclusion that increasing *d* = *d*_1_ = *d*_2_ will increase ∂(*X*_1_*+ *X*_2_*)/∂*E*.

For predator-prey interactions, equation (S8) also applies, although the signs of the terms differ from the cases of competition and mutualism. The term *d*_1_ represents the cross derivatives [D_2_α_1_] and [D_12_α_1_], and similarly *d*_2_ represents [D_1_α_2_] and [D_12_α_2_]. Because there is only a single function giving the predation rate, α_1_(υ_1_, υ_2_) = *a*(υ_1_, υ_2_), and α_2_(υ_1_, υ_2_) = –*a*(υ_1_, υ_2_)*c*, so *d*_1_ = –*cd*_2_. For traits to have optimal values, *a*_33_ < 0 and *a*_44_ < 0. In contrast to competition and mutualism, *a*_23_*a*_32_ ≤ 0 and *a*_14_*a*_41_ ≥ 0. Thus, positive values of *d* = *d*_1_ = –*cd*_2_, which will be the case for predator-prey relationships, will in general decrease ∂(*X*_1_*+ *X*_2_*)/∂*E*. Although this analysis only addresses the case when the environmental change *E* affects the prey intrinsic rate of increase, a symmetric analysis shows similar results for the case of *E* affecting predators.
